# Higher Prevalence and Abundance of *Bdellovibrio bacteriovorus* in the Human Gut of Healthy Subjects

**DOI:** 10.1371/journal.pone.0061608

**Published:** 2013-04-16

**Authors:** Valerio Iebba, Floriana Santangelo, Valentina Totino, Mauro Nicoletti, Antonella Gagliardi, Riccardo Valerio De Biase, Salvatore Cucchiara, Lucia Nencioni, Maria Pia Conte, Serena Schippa

**Affiliations:** 1 Department of Public Health and Infectious Diseases, ‘Sapienza’ University of Rome, Rome, Italy; 2 Regional Cystic Fibrosis Centre, ‘Sapienza’ University of Rome, Rome, Italy; 3 Department of Paediatrics and Infant Neuropsychiatry, ‘Sapienza’ University of Rome, Rome, Italy; 4 Department of Biomedical Sciences, University of Chieti ‘G. D’Annunzio’, Chieti, Italy; Charité, Campus Benjamin Franklin, Germany

## Abstract

**Introduction:**

Members of the human intestinal microbiota are key players in maintaining human health. Alterations in the composition of gut microbial community (dysbiosis) have been linked with important human diseases. Understanding the underlying processes that control community structure, including the bacterial interactions within the microbiota itself, is essential. *Bdellovibrio bacteriovorus* is a gram-negative bacterium that preys other gram-negative species for survival, acting as a population-balancer. It was found in terrestrial/aquatic ecosystems, and in animal intestines, postulating its presence also in the human gut.

**Methods:**

The present study was aimed to evaluate, by end-point PCR and qPCR, the presence of *B. bacteriovorus* in intestinal and faecal biopsy specimens from 92 paediatric healthy subjects and patients, suffering from Inflammatory Bowel Diseases (IBD), Celiac disease and Cystic fibrosis (CF).

**Results:**

i) *B. bacteriovorus* was present and abundant only in healthy individuals, while it was heavily reduced in patients, as in the case of IBD and Celiac, while in CF patients and relative controls we observed comparable results; ii) *B. bacteriovorus* seemed to be mucosa-associated, because all IBD and Celiac biopsies (and related controls) were treated with mucus-removing agents, leaving only the mucosa-attached microflora; iii) *B. bacteriovorus* abundance was district-dependent, with a major preponderance in duodenum, and gradually decreasing up to rectum; iv) *B. bacteriovorus* levels significantly dropped in disease status, in duodenum and ileum.

**Conclusions:**

Results obtained in this study could represent the first step for new therapeutic strategies aimed to restore a balance in the intestinal ecosystem, utilizing *Bdellovibrio* as a probiotic.

## Introduction

The largest collection of microbes resides in our gut, which harbours trillions of bacteria [1]. Although these populations are highly stable, they are still prone to perturbations by environmental insults [2], with important consequences for our physiology and, consequently, our health. Shaped by millennia of co-evolution, host and bacteria have developed beneficial relationships, creating an environment for mutualism, were the mutual survival of harboured microbiota and human host is interdependent [3], [4] **.** Alterations in the development or composition of the microbiota could affect the cross-talk between microbiota and host compromising human health. Microbiota ecosystem unbalance, named dysbiosis, has been found in different pathologies such as Inflammatory Bowel Diseases (IBD) [5]–[8], Celiac disease (CD) [9], [10] and Cystic fibrosis (CF) [11]. In IBD patients, for example, it has been showed a decrease in prevalence of members of the human commensal microbiota (i.e. *Clostridium* IXa and IV groups, *Bacteroides, Bifidobacteria*) and a concomitant increase in detrimental bacteria (i.e. sulphate-reducing bacteria, *Escherichia coli*) [1], [5], [12]. In patients with susceptible genetics the gut microbiota could drive the expansion of ‘pro-inflammatory’ species and the restriction of the protective/regulating species. Although the factors driving the composition dynamics of resident microbial communities are not well defined, understanding the mechanisms and relationships that control the delicate balance among the microbial groups is of primary importance. Bacteria, in their natural environments, are subjected to predation from bacteriophages, protists and predatory prokaryotes. *Bdellovibrio bacteriovorus* is a predatory gram-negative bacterium belonging to the δ-proteobacteriaceae sub-family, which attacks other gram-negative bacteria and invade their periplasm, using them as substrates for growth and reproduction [13]. This particular intracellular niche allows *B. bacteriovorus* to feed without competition and, through its high lytic capability, can rapidly reduce gram-negative populations. From its discovery in 1963 [14], *B. bacteriovorus* was under investigation for decades, but only in recent years it took the stage [15]. Its life cycle consists of two major steps: a ‘free-swimming/gliding’ one spent searching for prey in water or soil, and a double ‘attack phase/growth’ stage spent inside the periplasm of the prey bacterium [16], [17]. Once the resources of the prey have been consumed, the *B. bacteriovorus* divides into progeny which then lyses the residue cells and swim away to chase new hosts [18]. Depending on the prey concentration and environment, this life cycle takes roughly 3–4 h. Due to its selective preying capacity on gram-negative bacterial species [19], [20], *B. bacteriovorus* is probably involved in keeping the balance between the different bacterial species living together in a community, acting as an ecological balancer [21]. For example, *Bdellovibrio* delivered orally to live birds caused an increase in some gram-positive bacteria, a reduction in gram-negative bacteria (like *Salmonella*) and a parallel reduction in mucosal inflammation, without affecting the bird health [22]. Moreover, *B. bacteriovorus* population levels respond to the Lotka-Volterra prey-predator oscillation [23], where great *B. bacteriovorus* expansions [24] are balanced by a phenotypic resistance of the prey [25]. Thus, also in the intestine, in which polimicrobial cohorts coexists [26]–[28], it could be expected that a similar self-regulation for this predatory bacterium [29]. *B. bacteriovorus* is ubiquitous in terrestrial and aquatic environments, even if at low counts [20], [30]–[32], and it has also been isolated from intestines of vertebrates [33]–[35], indicating that it is probably present in the human gut [36]. The hypothesis underlying this study was that a dysbiotic microbial community, as found in inflammatory diseases, could be linked to differential prevalence and abundance of bacterial species acting as ecologic equalizer, such as *B. bacteriovorus*. To this purpose, we evaluated by PCR the presence of the bacterial predator *B. bacteriovorus* in bioptic samples collected from IBD and Celiac patients, as well as in faecal samples taken from Cystic fibrosis patients. Sex- and age- matched controls were also enrolled to compare the prevalence and relative abundance of *B. bacteriovorus* in the healthy status.

## Materials and Methods

### Ethics Statement

Intestinal biopsies were obtained from gastro-duodenoscopy (for ‘Celiac group’) and colonoscopy (for ‘IBD group’) procedures carried out at the ‘Policlinico Umberto I’ Hospital at Rome, Italy. Ethics approval for this study was granted by the Ethics Committees of the ‘Sapienza’ University and ‘Policlinico Umberto I’ Hospital, Italy. Written informed consent was obtained from parents of all subjects enrolled in this study who were under 18 years of age, while the written informed consent was given autonomously by subjects over (or equal to) 18 years. In any case, written informed consent was obtained upon instructions on ethics, aims, and methodologies employed in the study.

### Patients

#### ‘Inflammatory bowel disease group’

Twenty-three paediatric patients referred to the Paediatric Gastroenterology and Liver Unit of the Sapienza University of Rome, Italy, for suspected IBD were studied: active CD was diagnosed in 9, and active UC was diagnosed in 6. The remaining 8 subjects with functional gastrointestinal disorders (lymphonodular hyperplasia), and normal colonoscopy and histology findings, served as controls. In table 1 were reported the patients’ baseline demographics. The patients’ groups did not differ significantly by age and disease duration. All children with CD had ileocolonic involvement, and all had disease activity in the moderate to severe range. All UC patients had endoscopic evidence of pancolitis, and the disease was classified severe. The diagnostic workup of UC and CD was done according to international protocols. Children with CD were assessed using the Paediatric Crohn’s Disease Activity Index (PCDAI), which is a multi-item score based on recall of the preceding week’s symptoms, laboratory parameters (erythrocyte sedimentation rate, haematocrit and albumin levels), and physical examination. A score of 10 implies inactive disease, one of 11 to 30 implies mild disease, and one of >30 implies moderate to severe disease. It is noteworthy that our CD paediatric patients did not have any previous endoscopic assessment (see table 1), nor any kind of treatment, so they had naïve mucosal inflammation. Patients with UC were evaluated using the Paediatric Ulcerative Colitis Activity Index (PUCAI), which is based only on clinical symptoms. A score of 10 indicates inactive disease, one of 11 to 34 implies mild disease, one of 35 to 64 implies moderate disease, and one greater than 65 implies severe disease. Patients within UC cohort had also a naïve mucosal inflammation. None of the children included in the study, either CD, UC and controls, was treated with antibiotics for at least 3 months before the sampling time. The study protocol was approved by the Committee on Ethical Practice of the ‘Sapienza’ University of Rome and ‘Policlinico Umberto I’ hospital, as reported in the ‘Ethics statement’. All paediatric patients underwent ileocolonoscopy after parental informed written consent was provided. During ileocolonoscopy, for routine histological assessment and bacteriological study, two biopsy samples were taken from each region (ileum, descending colon, rectum), in a non-inflamed patch adjacent to an inflamed one. Specimens were collected in 2-ml screw-cap vials filled with 0.85 ml of brain heart infusion broth (Oxoid, Cambridge, United Kingdom) and 0.15 ml of glycerol (Sigma-Aldrich, St. Louis, MO) and immediately stored at −80°C.

**Table 1:** Demographics and baseline disease characteristics of the patients’ groups.

	Crohn’s Disease	Ulcerative Colitis	Controls	Celiac Disease	Controls	Cystic Fibrosis	Controls
No. of cases	9	6	8	10	8	35	16
Age (years; mean ± SD)	14.0±2.5	12.0±4.1	12.7±1.8	8.3±3.7	11.7±3.5	18.6±2.2	16.3±1.7
M/F	4/5	3/3	4/4	5/5	4/4	18/17	8/8
Weight (Kg) (mean ± SD)	30.6±12.2	33.9±13.1	36.2±9.6	29.7±3.2	40.9±3.2	45.2±2.4	48.7±5.6
Height (cm) (mean ± SD)	133.8±19.1	138.3±22.1	140±25.3	128.9±5.5	147.9±3.7	150.5±2.9	159.2±6.2
BMI (Kg/m^2^) (mean ± SD)	16.4±2.0	17.0±2.2	20.0±2.1	16.7±0.5	18.5±0.8	19.4±0.5	19.8±0.4
Disease duration (weeks; median and range)	6 (2–12)	8 (3–12)	–	36 (32–56)	–	–	–
Duodenum involvement(a)	–	–	–	10/10 (100%)	–	–	–
Ileo-colon involvement	9/9 (100%)	0/6 (0%)	–	–	–	–	–
Colon involvement	0/9 (0%)	6/6 (100%)	–	–	–	–	–
Rectum involvement	0/9 (0%)	6/6 (100%)	–	–	–	–	–
Previous endoscopic assessment	0/9 (0%)	0/6 (0%)	0/8 (0%)	0/10 (0%)	0/8 (0%)	–	–
Current treatment (b)	0/9 (0%)	0/6 (0%)	0/8 (0%)	0/10 (0%)	0/8 (0%)	35/35 (100%)	–
PCDAI (mean ± SD)(c)	42.5±11	–	–	–	–	–	–
PUCAI (mean ± SD) (c)	–	71.4±7.5	–	–	–	–	–
Marsh index	–	–	–	IIIA 3/10 (30%); IIIB 3/10 (30%); IIIC 4/10 (40%)	–	–	–
FEV1%(c)	–	–	–	–	–	91.8±2.6	–

(a) Biopsies were taken in the second part of duodenum.

(b) IBD: Sulfasalazine, 5-aminosalicylic acid drug, Steroid; CF: ciproxin 500 mg (x2)/die (not provided for 2 months prior sampling).

(c) PCDAI, Paediatric Crohn’s Disease Activity Index; PUCAI: Paediatric Ulcerative Colitis Activity Index; FEV1%, Forced Expiratory Volume (in percentage).

#### ‘Celiac disease group’

Two groups of children referred to the Paediatric Gastroenterology and Liver Unit of the “Sapienza” University of Rome were included in this study: 10 Celiac patients in active state and 8 controls undergoing upper gastrointestinal endoscopy for functional dyspepsia. Controls tested negative for antitransglutaminase and antiendomysial antibodies with normal IgA levels, while histology of duodenum did not reveal features of Celiac disease. Diagnosis of Celiac disease had been performed according to ESPGHAN criteria [37], and table 1 summarizes clinical features of the studied population. It is noteworthy that our CD paediatric patients did not have any previous endoscopic assessment (see table 1), nor any kind of treatment, so they had naïve mucosal inflammation. Size-appropriated and well-oriented endoscopic biopsy specimens were obtained from the second part of the duodenum, in a non-inflamed patch adjacent to an inflamed one. The histopathological diagnosis (Marsh index) was based on typical mucosal lesions with crypt cell hyperplasia, villous atrophy, and increased number of intra-epithelial lymphocytes (IELs) [38]. All untreated Celiac patients were positive for antiendomysial and antitransglutaminase antibodies at the time of diagnosis. None of the children included in the study was treated with antibiotics for at least 3 months before the sampling time. The study protocol was approved by the Committee on Ethical Practice of the ‘Sapienza’ University of Rome and ‘Policlinico Umberto I’ hospital, as reported in the ‘Ethics statement’. Children were enrolled in the study after written informed consent from their parents. Specimens were collected in 2-ml screw-cap vials filled with 0.85 ml of brain heart infusion broth (Oxoid, Cambridge, United Kingdom) and 0.15 ml of glycerol (Sigma-Aldrich, St. Louis, MO) and immediately stored at −80°C.

#### ‘Cystic fibrosis group’

Thirty-five CF patients, referred to the Cystic Fibrosis Centre of the Department of Paediatrics at Hospital ‘Policlinico Umberto I’ of Rome, were enrolled after written informed consent. Sixteen age- and sex-matched controls were also enrolled. The diagnostic work up of CF was according to international protocols. The study protocol was approved by the Committee on Ethical Practice of the ‘Sapienza’ University of Rome and ‘Policlinico Umberto I’ hospital, as reported in ‘Ethics statement’. All patients did not received antibiotics during 2 months prior to the beginning of the study, and were fasting prior faecal sampling. Faecal samples were collected in a 50 mL tube, and immediately frozen at −80°C.

### Biopsy DNA Extraction

Biopsy specimens were taken from fasting patients (Crohn’s disease, Ulcerative colitis, Celiac disease) and proper age−/sex-matched controls by the same gastro-endoscopist, and immediately stored in 2 mL tubes at −80°C, as described above. Within 2 hours from sampling, biopsies were first quickly washed three times in 500 µL of physiologic saline with 0.016% dithiothreitol (DTT) to remove luminal bacteria and the mucus layer, and then utilized for DNA extraction procedure by DNeasy tissue kit (Qiagen, Hilden, Germany) according to the manufacturer’s instructions. In order to obtain maximum yield of Gram-negative bacteria, a special step in DNA purification protocol was added, following DNeasy tissue kit manual. Briefly, 180 µL of ATL buffer were added to sample followed by 180 µL volume of enzymatic lysis buffer (20 mM Tris·Cl, pH 8.0, 2 mM sodium EDTA, 1.2% Triton X-100, lysozyme to 20 mg/ml), and incubated for 30 minutes at 37°C. Next, 25 µL of proteinase K solution and 200 µL of buffer AL were added, followed by an incubation step at 56°C for 30 minutes. DNA concentration was determined using a NanoDrop spectrophotometer (Thermo Fisher Scientific, Wilmington, Delaware, U.S.A.) at 260 nm. The 260/280 ratio was also measured to ensure a proper DNA purity. We obtained similar DNA concentrations after kit extraction both from patients and controls biopsies: a Mann-Whitney U test was performed on total DNA concentration (*P* = 0.117), indicating a similar amount of extracted DNA in all the cohorts (‘IBD group’ and ‘Celiac group’).

### Faecal DNA Extraction

Faecal samples were taken from fasting CF patients and age- and sex-matched controls in a sterile environment placed into the ‘Policlinico Umberto I’ hospital, collected in a 50 mL tube, and immediately frozen at −80°C. Total DNA was extracted within 1 hour from sampling by QIAmp Stool Mini Kit (QIAGEN, Hilden,Germany) following manufacturer’s instructions. Starting faecal amount was set at 500 mg, picked up from different chunks within the sample itself, in order to minimize the sampling error. Upon extraction, DNA concentration was quantified by a NanoDrop spectrophotometer (Thermo Fisher Scientific, Wilmington, Delaware, U.S.A.) at 260 nm, along with the 260/280 ratio, and integrity checked through 1% agarose gel electrophoresis containing EtBr 0.5 µg/ml. We obtained similar DNA concentrations after kit extraction both from CF patients and controls biopsies. A Mann-Whitney U test was performed on total DNA concentration (*P* = 0.348), indicating a similar amount of extracted DNA in both cohorts within the ‘CF group’.

### *B. bacteriovorus* Cultivation and DNA Extraction

The predatory bacteria used was *B. bacteriovorus* strain HD 100 (DSM No.: 12732) taken from Deutsche Sammlung von Mikroorganismen und Zellkulturen (DSMZ, Braunschweig, Germany), maintained as plaques in double-layered diluted nutrient broth (DNB) agar, a 1∶10 dilution of nutrient broth amended with 3 mM of MgCl_2_·6H_2_O and 2 mM of CaCl2·2H2O [pH 7.2] and agar (0.6% agar in the top layer) [39]. To initiate a lysate, co-cultures were obtained by adding a plug of agar containing *B. bacteriovorus* plaque to washed prey cells in DNB and incubated at 30°C on a rotary shaker set at 200 rpm until the co-culture became clear (stock lysate). To harvest the predators, co-cultures were prepared in which 2 ml of washed host cells (10^9^ CFU/ml) was incubated with 2 ml of stock lysate in 20 ml of DNB. The co-cultures were incubated for 18 h to reach a final concentration of 10^8^ plaque-forming units (PFU)/ml of predator. At this point, the lysate was passed three times through a 0.45-µm Millex pore-size filter (Millipore, Billerica, MA, USA) to remove residual prey and cell debris (filtered lysate). As a control, filtered sterilized lysate was prepared by sequentially passing the lysates through three 0.22-µm pore-size filters. After filtration, no predator, as judged by PFU, could be detected [40]. DNA was extracted from the 0.45-µm-filtered suspension of *B. bacteriovorus* through thermal lysis, and used in subsequent PCR as positive control.

### PCR Procedure Work-flow

The present study was aimed to evaluate, by PCR technique, the presence of *B. bacteriovorus* in intestinal and faecal biopsy specimens from paediatric healthy subjects and patients suffering from IBD, Celiac disease and CF. To this purpose, we carried out the following PCR reactions: i) ‘β-globin gene amplification’ on the total DNA extracted from all samples collected, in order to verify DNA suitability for amplification; ii) *Bdellovibrio* species-specific end-point PCR and qPCR on the total DNA extracted from all samples collected, in order to verify the *Bdellovibrio* presence; iii) 16S rDNA amplification on negative *Bdellovibrio* species-specific PCR samples, in order to asses if overall bacterial DNA was present in such samples, thus ensuring their negativity for *Bdellovibrio*.

### Detection of *B. bacteriovorus* in Intestinal Biopsies and Faecal Samples by Species-specific PCR

A first-step assessment of DNA suitability for subsequent *B. bacteriovorus*-specific PCR was achieved through a human β-globin gene amplification for each sample, as described previously [41]. Briefly, aliquots of each DNA sample (50 ng) were amplified with specific primers: forward primer, 5′-CAACTTCATCCACGTTCACC-3′; reverse primer, 5′-GAAGAGCCAAGGACAGGTAC-3′. Amplification reactions were carried out in a 50-µl volume containing 1× PCR buffer II (Applied Biosystems, Roche, California, USA), 3 mM magnesium chloride, 200 µM each deoxynucleoside triphosphate, 50 pmol each primer and 1.5 U of AmpliTaq Gold polymerase (Applied Biosystems). The PCR was carried out under the following conditions: 1 cycle of 95°C for 7 min, 35 cycles of 94°C for 1 min, 55°C for 1 min and 72°C for 1 min and 1 cycle of 72°C for 7 min. Twenty microliters of each PCR were run on a 1% agarose gel containing EtBr 0.5 µg/ml, and photographed with DigiDoc-It system (UVP, Cambridge, UK).

*B. bacteriovorus* specific primers Bd529F (5′-GGTAAGACGAGGGATCCT-3′) and Bd1007R (5′-TCTTCCAGTACATGTCAAG-3′) were used to amplify a 481-bp trait of the 16S rDNA gene [31]. At the same time, primers targeting a 910-bp trait of the *hit* locus, specific for *B. bacteriovorus* [36], [42], were employed: Hit_FW (5′-GACAGATGGGATTACTGTCTTCC-3′) and Hit_RW (5′-GTGTGATGACGACTGTGAACGG-3′). We used these two sets of primers because they are reported to be specific for *B. bacteriovorus* PCR amplification from total DNA, and we decided to use them both to strength the results. PCR was performed with Taq DNA-polymerase (Hot Star Taq Plus, QIAGEN, Hilden, Germany). PCR reaction (25 µl) contained 1X buffer for PCR, 300 µg/mL bovine serum albumin (Sigma-Aldrich, St. Louis, Missouri, United States), 2.5 mM MgCl2, 200 µM for each dNTP, 0.5 µM of each primer, 1.25 U of Taq polymerase and 100 ng of total DNA (from biopsies or faeces). For 16S rDNA trait, sample DNA was amplified under the following conditions: 95°C for 5 min, 20 cycles of 95°C for 1′, 53°C for 1′, 72°C for 1′, and a final step of 72°C for 10 minutes. For *hit* locus, sample DNA was amplified under the following conditions: 95°C for 5 minutes, 20 cycles of 95°C for 1′, 60°C for 1′, 72°C for 1′, and a final step of 72°C for 10 minutes. Appropriate positive (*B. bacteriovorus* strain HD100) and negative (water) controls were employed.

In order to minimize the PCR bias, and to avoid the problem due to the expected low levels of *B. bacteriovorus* in samples, we performed three individual PCR reactions for each sample. The individual PCR reactions were unified, analysed by electrophoresis on 2% agarose gels containing ethidium bromide to determine their size (481 bp), and concentrated with SpeedVac (Savant, Holbrook, NY, USA) to reach a final volume approximately equal to 1/3 of the original. The unified PCR reactions were titrated using two different methods: firstly, twenty-five microliters of each concentrated PCR were loaded on a 1% agarose gel containing EtBr 0.5 µg/ml, run for 1 hour at 80 V, photographed with DigiDoc-It system (UVP, Cambridge, UK), and analysed for densitometry with Phoretix 1D software (TotalLab, Newcastle upon Tyne, United Kingdom); secondly, measure of DNA density was performed by NanoDrop spectrophotometer (Thermo Fisher Scientific, Wilmington, Delaware, U.S.A.) at 260 nm, using one microliter of unified PCR. The results obtained by such measures were in agreement one each other and, upon normalization, were used for subsequent analyses.

All positive PCR products were sequenced using the BigDyeTM terminator chemistry (Applied Biosystems, Foster City, USA) and the sequencing mixture was analysed on a DNA sequence analyser ABI3730 (Applied Biosystems, Foster City, USA). Sequencing was performed in both directions and sequences were analysed with SEQ MATCH at the Ribosomal Database Project II website (http://rdp.cme.msu.edu/seqmatch/seqmatch_intro.jsp). The obtained sequences were also compared to gene sequences of known bacterial identities available in GenBank by means of the Basic Local Alignment Search Tool (BLAST) present at National Centre for Biotechnology Information (NCBI) website (http://www.ncbi.nlm.nih.gov).

### *In silico* PCR Amplification

To ensure that both primer pairs used, for 16S rDNA trait and *hit* locus, were targeted towards *B. bacteriovorus* genome, we performed an *in silico* PCR method through the online tool present at the website of the University of the Basque Country, employing the sequenced genome of the strain HD100: http://insilico.ehu.es/PCR/index.php?mo=Bdellovibrio. Such an approach allowed us to determine that *B. bacteriovorus* has two 16S rDNA operons (first target, from 820075 bp to 820555 bp, and second target, from 1688139 bp to 1688619 bp), while it harbours only one *hit* locus on the reverse strand (from 96861 bp to 97770 bp). The information so obtained was used to properly normalize the relative abundances of *B. bacteriovorus*.

### Bacterial 16S rDNA Amplification with Universal Primers on Negative PCR Samples

In order to confirm the significance of results, a further 16S rDNA PCR was performed on all *B. bacteriovorus* PCR-negative samples, detecting the presence of bacterial DNA. Universal primers U968 (5′-GAA CGC GAA GAA CCT TAC-3′) and L1401 (5′-GCG TGT GTA CAA GAC CC-3′) were used to amplify the V6–V8 region of bacterial gene coding for 16S rRNA. PCR was performed with Taq DNA-polymerase (Hot Star Taq Plus, QIAGEN). PCR reaction (25 µl) contained 1X buffer per PCR, 2.5 mM MgCl_2_, 200 µM for each dNTP, 0.5 µM of U968-GC and L1401 oligos, 1.25 U of Taq polymerase and 100 ng of total DNA. The samples were amplified under the following conditions: 95°C for 5 min, cycles at 94°C for 45″, 53°C for 45″, 72°C for one min and 10″, and a final step of 72°C for 30 min. To rule out non-specific products, a ‘touchdown PCR’ was performed with a starting annealing temperature of 58°C and decreasing it by 0.5°C each cycle to reach 53°C, followed by 30 cycles at 53°C. In order to minimize the PCR bias, three ‘touchdown PCR’ reactions were performed for each sample and subsequently pooled. To minimize hetero-duplex formation and single-stranded DNA (ssDNA) contamination during PCR amplification, 5 additional cycles of ‘reconditioning PCR’ were performed, taking 1/10 of the previous pooled PCR volume as template in a new reaction. In order to minimize the PCR bias, three ‘reconditioning PCR’ reaction were done for each sample and subsequently pooled. Successful reaction and DNA concentration was quantified by spectrophotometer measurements at 260 nm and DNA integrity checked through 1% agarose gel electrophoresis containing EtBr 0.5 µg/ml.

### Quantitative-PCR

We performed a quantitative PCR (qPCR) approach with standard curve in order to assay the relative abundances of *B. bacteriovorus* in our samples. Primers and hydrolysis probe used were taken from the literature [43], amplifying a *B. bacteriovorus*-specific region of 16S rDNA. Briefly, primers used were Bd347F (3′-GGAGGCAGCAGTAGGGAATA-5′) and Bd549R (5′-GCTAGGATCCCTCGTCTTACC-3′), while the probe was Bb396P (5′FAM-TTCATCACTCACGCGGCGTC-TAMRA3′). The qPCR reaction mix was made up with 10 µl of ‘2X qPCR ProbesMaster’ from Jena Bioscience GmbH (Jena, Germany) (composition: qPCR Pol, dATP, dCTP, dGTP, dUTP, reaction buffer with KCl, (NH_4_)_2_SO_4_ and MgCl_2_, ROX, stabilizers), 900 nM for both of the primers, 50 nM for the probe, and 5 µl of template (corresponding to 200 ng of total DNA). The final reaction volume was adjusted to 20 µl with PCR-grade distilled water. Thermal cycling conditions were: 2 min at 95°C (initial denaturation), followed by 50 repeats of 15 s at 95°C (denaturation) and 1 min at 60°C (annealing and extension). Data were collected during the annealing phase. Three different replicates were performed for each sample. In each run no template controls (NTC) were included, along with positive controls. To construct the standard curve, a plasmid (pUC57) containing one copy of the target was provided by Biofab Research (Rome, Italy), quantified by spectroscopy at 260 nm, and 10-fold serially diluted in the range of 10^9^-10^0^ copies [44]. Results of qPCR were normalized for the number of 16S rDNA operons within *B. bacteriovorus* genome [43], and expressed as ‘number of genome copies/mg of sample’ (biopsies or faeces), following the Minimum Information for publication of Quantitative real-time PCR Experiments (MIQE) guidelines [45].

### Statistical Methods

To assess putative differences among and within the patients’ groups (IBD, Celiac, CF) in terms of prevalence of *B. bacteriovorus* in subjects and samples, we used the χ^2^ test. To assess significant differences among the aforementioned groups in terms of relative abundance of *B. bacteriovorus* in samples, a Mann-Whitney U test was employed. For both tests, a *P* value less than or equal to 0.05 was considered significant.

## Results

### *B. bacteriovorus* Prevalence in Subjects and Samples

We analysed overall 87 intestinal biopsies from healthy controls (n = 8) and patients affected by Crohn’s (n = 9), Ulcerative colitis (n = 6) and Celiac disease (n = 10), and 51 faecal samples from healthy controls (n = 16) and Cystic fibrosis patients (n = 35). Species-specific end-point PCRs and qPCR were employed to detect the presence of *B. bacteriovorus* in the aforementioned patients. All end-point PCR-positive reactions were sequenced and submitted to SEQ MATCH and BLAST, finding a percentage similarity of 100% against *B. bacteriovorus* strain HD100 for all the sequences. Specifically, the percentage similarity was 100% across 481 bp for 16S rDNA locus, while it was 100% across 910 bp for *hit* locus. We found reliable results among the two sets of primers used.

**Figure 1:**
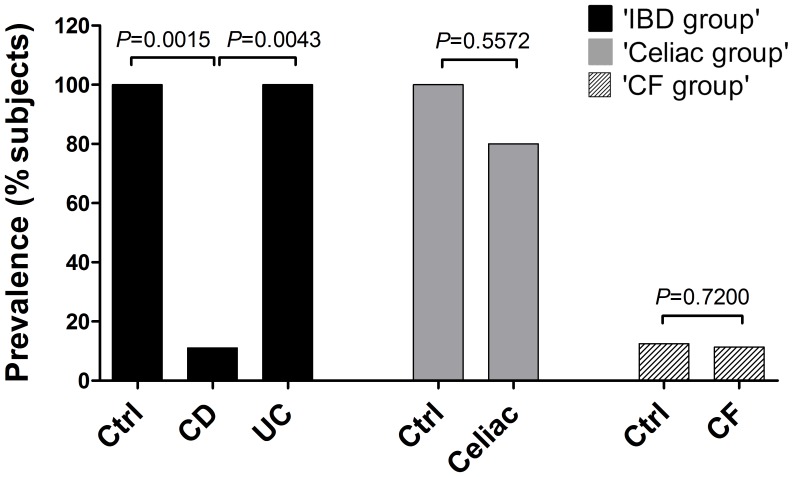
Prevalence of *B. bacteriovorus* in subjects from IBD, Celiac, and CF groups. Ninety-two subjects were enrolled: 23 within ‘IBD group’ (9 CD, 6 UC, 8 controls), 18 within ‘Celiac group’ (10 Celiac, 8 controls), and 51 within ‘CF group’ (35 CF, 16 controls). Bars represent the relative prevalence of *B. bacteriovorus* among subjects. *P* values were computed through χ^2^ test.

We had reliable results among the two approaches used to detect *B. bacteriovorus* prevalence. Within the ‘IBD group’, *B. bacteriovorus* was significantly found in 8/8 (100.0%) of control subjects, in 6/6 (100%) of UC patients, and in 1/9 (11.1%) of CD patients. Within the ‘Celiac disease group’, *B. bacteriovorus* was found in 8/8 (100%) of control subjects, and in 8/10 (80.0%) of Celiac patients. Within the ‘CF group’, it was found in 2/16 (12.5%) of controls, and in 4/35 (11.4%) of CF patients, with no significant difference. These results, together with *P* values, are summarized in figure 1.

Results of *B. bacteriovorus* prevalence in biopsy samples of the ‘IBD group’, taken within a single subject from different districts, were reported in table 2. Prevalence of *B. bacteriovorus* was assessed taking into account both end-point and qPCR data. We observed a preponderant prevalence of *B. bacteriovorus* in ileum, colon, and rectum of control biopsies (20/24, 83.3%) in respect to CD (1/27, 3.7%, *P*<0.0001). No difference was found among overall UC (15/18, 83.3%) and control samples *P* = 0.6760). It is noteworthy that all ‘IBD group’ biopsy samples (ileum, colon, rectum) were treated with the reducing agent dithiothreitol (DTT), in order to remove the mucus layer and the luminal flora, as reported in Materials and Methods section. Thus, the mucosa-associated flora was retained, along with mucosa-associated *B. bacteriovorus*.

**Table 2:** Prevalence of *B. bacteriovorus* (% of positive samples) in the ‘IBD group’.

	CD (n = 27)	UC (n = 18)	Controls (n = 24)
Ileum	0/9 (0)	5/6 (83.3)	7/8 (87.5)
Colon	1/9 (11.1)	5/6 (83.3)	7/8 (87.5)
Rectum	0/9 (0)	5/6 (83.3)	6/8 (75)

Numbers within brackets are percentage values.

### *B. bacteriovorus* Relative Abundance in Subjects and Samples Assessed by qPCR

Data about *B. bacteriovorus* abundance were collected by qPCR. Within-cohort mean values, expressed as number of copies/mg of sample (biopsies or faeces), were compared after a proper correction for the number of 16S operons, namely two in *B. bacteriovorus* genome, as assessed by *in silico* PCR. Standard curves gave a mean slope of −3.3392±0.0405, a mean intercept of 39.4547±0.3976, and a mean R^2^ equal to 0.9970±0.0007 (Figure S1). The mean reaction efficiency (E), calculated from the mean value of the slope by means of the equation E = 10^(−1/slope)^, was 99.4% ±1.6%. The lowest reproducible detection level of the qPCR was 10 plasmids per reaction, each containing one target sequence.

**Figure 2:**
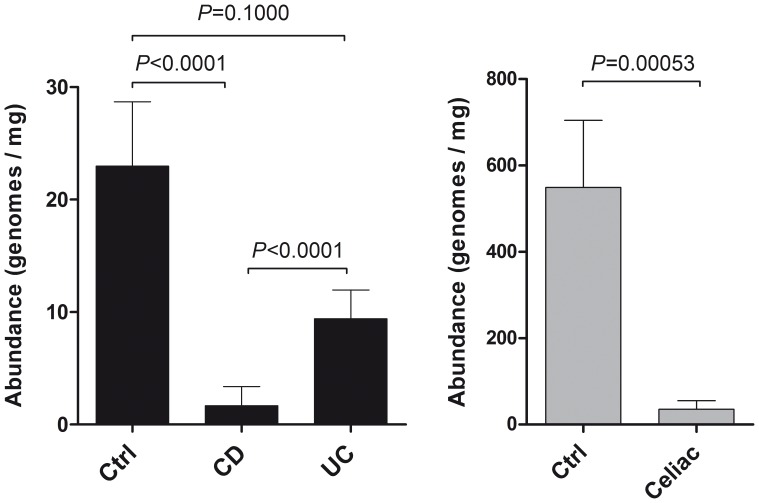
Relative abundance of *B. bacteriovorus* in samples from IBD and Celiac groups. In total, 138 samples were taken into account: 69 intestinal biopsies from ‘IBD group’ (27 CD, 18 UC, 24 controls), 18 within ‘Celiac group’ (10 Celiac, 8 controls), and 51 faecal samples within ‘CF group’ (35 CF, 16 controls). Differences in relative abundances were assessed by Mann-Whitney U test.

Results of qPCR were: 549±156 copies/mg and 35±20 copies/mg, for controls *vs* Celiac; 23±6 copies/mg, 2±2 copies/mg, and 9±2 copies/mg for controls, CD and UC. In faecal samples of ‘CF group’, *B. bacteriovorus* abundance was always under the limit of detection in both controls and CF patients. We found that relative abundance of *B. bacteriovorus* was significantly higher in control bioptic samples of both ‘IBD group’ and ‘Celiac group’ (Figure 2). In particular, significant differences in relative abundances were found among controls *vs* CD (*P*<0.0001), UC *vs* CD (*P*<0.0001), and controls *vs* Celiac (*P* = 0.00053). In UC, even though *B. bacteriovorus* is always present (Figure 1), we observed a drop in its relative abundance of 59.1% in respect to controls (Figure 2). Also in Celiac patients we found an important presence of *B. bacteriovorus* (80%) similar to controls (100%), but, contrarily, the 93.5% drop in its relative abundance was highly significant (*P*<0.0005) (Figure 2).

### *B. bacteriovorus* Relative Abundance is District-dependent

**Figure 3:**
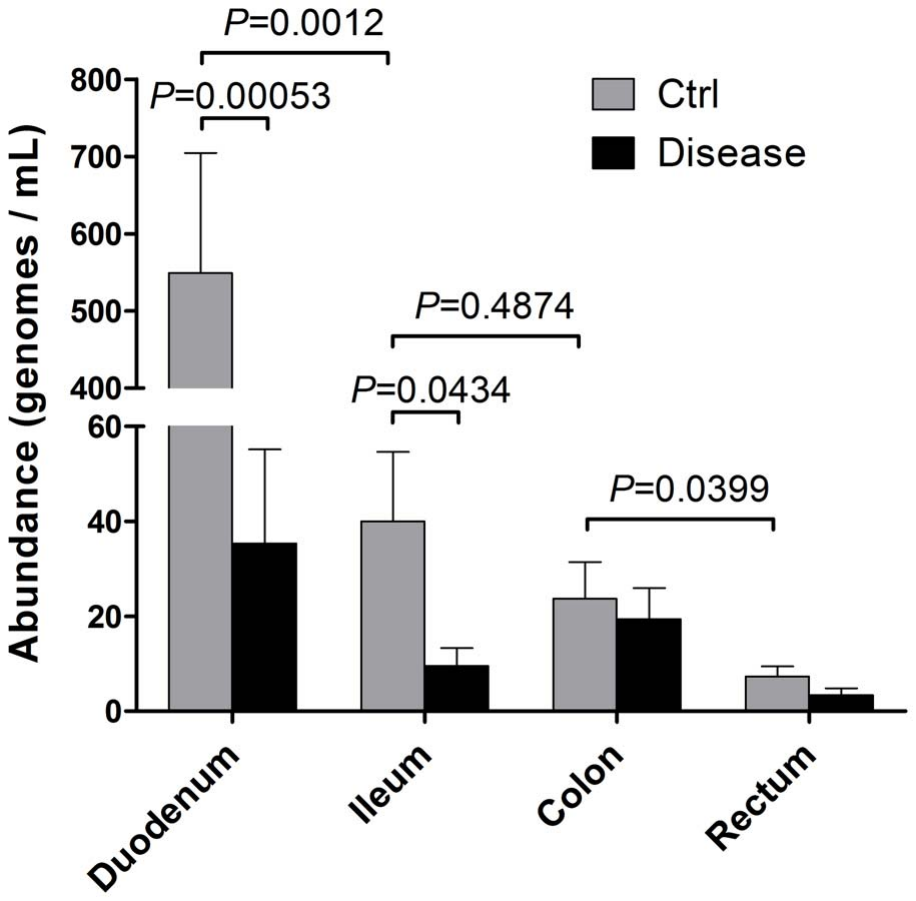
Longitudinal distribution of *B. bacteriovorus* along the intestine. We took into account samples from controls (grey bars) and diseased (black bars) subjects within each group. Bars represent the mean relative abundance of *B. bacteriovorus* found in these samples, divided by intestinal districts. *P* values were assessed by Mann-Whitney U test.

In order to analyse the relative abundance of *B. bacteriovorus* in different intestinal districts, we compared qPCR results obtained from ileum, colon, and rectum biopsy samples, and from faeces encompassing luminal flora. Results from controls clearly showed a decreasing trend of *B. bacteriovorus* relative abundance along the intestine (Figure 3, grey bars), in a cephalic-caudal direction. Interestingly, we observed the highest *B. bacteriovorus* levels in duodenum (*P* = 0.0012), and the lowest in rectum, while it was under the lower limit of qPCR detection in faeces. Furthermore, results in figure 3 evidenced a kind of ‘discrete distribution’ of B. *bacteriovorus* abundances, recognizing three different levels: starting from 10^2^ copies/mg in duodenum, 10^1^ in ileum/colon, we arrived at an order of 10^0^ in rectum.

In order to compare biopsy samples from controls and diseased subjects, mostly UC samples were taken into account, because CD samples resulted all *B. bacteriovorus*-negative (except one, a colonic biopsy). Comparing control and disease statuses (Celiac, duodenum; UC, ileum, colon, rectum), we observed a significant difference only in duodenum (*P* = 0.00053) and ileum districts (*P* = 0.0434) (Figure 3). We didn’t observe such a difference among control and disease status in colon (*P* = 0.7721) or rectum (*P* = 0.2432) districts.

## Discussion

This is the first extensive survey of *B. bacteriovorus* presence in humans, both in healthy and disease status. A precedent study [36] reported *B. bacteriovorus* presence in one human faecal sample. The occurrence of these predatory bacteria in the intestinal tract raises questions about their role in this habitat. Predation is an important mechanism in nature to keep bacterial populations under control and plays a major role in the cycling of nutrients through the microbial loop [46]. Predatory bacteria have been found, always at low population levels, in almost all prey-containing niches, ranging from marine sediments to fresh water and animal intestines. Our study hypothesis was that an imbalanced microbial community, such as observed in different diseases like IBD, Celiac disease and Cystic fibrosis, goes along with a loss/reduction of bacterial species acting as ecologic equalizer, such as *B. bacteriovorus*. We assessed *B. bacteriovorus* presence by end-point and qPCR. Our results showed that *B. bacteriovorus* is significantly present in paediatric healthy subjects and almost completely absent in CD and Celiac patients. Moreover, we found a significant difference among CD and UC for *B. bacteriovorus* prevalence in subjects (Figure 1), while UC and controls were similar. However, UC samples harboured a diminished *B. bacteriovorus* relative abundance (59.1%) in respect to controls. These last results could underscore the difference between CD and UC patients in terms of microbiota composition, and it could support the idea that UC patients are closer to controls in terms of intestinal habitat, in agreement with already reported studies [12], [47], [48].

As reported in figure 3, we found a differential cephalic-caudal distribution of *B. bacteriovorus* levels along the intestine in controls. The preferential duodenal colonization could reflect its predatory nature, or the existence of particular habitat conditions (pH, redox state, pO_2_, shear-stress). Duodenal district is usually colonized by a bacterial load in the order of 10^2^ CFU/ml, and our finding of a triple-level abundance of *B. bacteriovorus* along the gut, with a 10-fold drop in ileo-colonic districts, should reflect the suitable conditions for prey/predator equilibrium, as stated by Lotka-Volterra equation [23], [29], principally in duodenal district. However, we found that *B. bacteriovorus* could strive also in ileo-colonic region at mucosal level, a low-pO_2_ district.

It was found how *Bdellovibrio* could survive in anaerobic conditions [22], thus enabling the colonization of gut districts with low pO_2_. Interestingly, disease status affected *B. bacteriovorus* relative abundance mainly in upper GI districts, such as duodenum and ileum. Future studies should describe the *B. bacteriovorus* distribution in the human gut in a fine-tune fashion, to better understand its role within the bacterial community. In this study, for the first time, we showed the presence of *B. bacteriovorus* at the intestinal mucosal level, indicating that this predatory bacterium could be a permanent inhabitant of the gut ecosystem. We didn’t find it in faecal samples at significant prevalence or abundance. We cannot exclude that treatment with DTT, a reducing agent that loosen the disulphide bonds among mucin proteins, ultimately eliminating the mucus layer, should have removed a portion of the *B. bacteriovorus* population from biopsies. Using DTT, we left only the mucosa-attached microbiota, along with the predators inside their gram-negative preys, and, possibly, with a little percentage of free-swimming *B. bacteriovorus*. The intestinal mucus layer is 40–240 µm thick [49] and it should be a good ‘hunting field’ for *B. bacteriovorus* to chase prey: its long polar flagellum [50], [51] drives a speed of around 160 µm/s, (in certain cases up to 400 µm/s), and this speed would be even higher under certain viscosity conditions, as found for other bacterial species [52]. Thus, mucus removal could have excluded from DNA extraction, and subsequent PCR detection, a major part of free-swimming *B. bacteriovorus*. In this view, future experiments should address the free-swimming/intra-periplasmic ratio of *B. bacteriovorus* population within a specific sample, both with and without DTT treatment, to deepen the knowledge about its relative abundances along the intestinal tract.

The mucosa-associated microbiota, due to its close proximity to the intestinal epithelium and the underlying mucosal immune system, is believed to play a main role in maintaining the homeostasis in healthy gut and in the inflammatory response in diseases. The key role of *B. bacteriovorus* in the gut microbial community is exerted through its selective gram-negative predatory activity, as found in terrestrial and aquatic ecosystems [20], [30]–[32]. The low abundance of *B. bacteriovorus* in the intestinal mucosa of IBD and Celiac patients could bring to an uncontrolled bacterial overgrowth, as already reported in these subjects, where an increased number of mucosa-associated bacteria was found [9], [10], [41], [53]. In conclusion, the low prevalence of *B. bacteriovorus* at mucosal level, in both IBD and Celiac paediatric patients, could support the idea that loss in microbiota biodiversity could also involve species, such as predatory *Bdellovibrio*, that are normally underrepresented, and that exert a role in regulating the bacterial population levels. A recent study found that all BALOs species accounts for 0.3%–0.7% of the bacterial soil community, with *Bdellovibrio* genus representing around 80% of BALOs [54].

Further studies will be necessary to improve our results, along with an increase in number of subjects to be enrolled. The present study, performed on naïve paediatric patients and controls, enabled us to investigate an untouched gut ecosystem, with no previous endoscopic assessments or pharmacological treatments, thus supporting the idea of an early gut colonization by *B. bacteriovorus* from the environment. In the future, it will be interesting to check the *B. bacteriovorus* presence also in adult controls and patients affected by dysbiotic diseases. Thus, *B. bacteriovorus* could be a good candidate for new therapy strategies, such as shown in birds [22], aimed to restore the ecosystem balance, and, ultimately, to control the dysbiotic events.

## Supporting Information

Figure S1 **Standard curve for qPCR.** Standard curves gave a mean slope of −3.3392±0.0405, a mean intercept of 39.4547±0.3976, and a mean R^2^ equal to 0.9970±0.0007. As an example, in figure is the standard curve relative to UC samples. On *x* axis, log of ‘number of plasmid copies’; on *y* axis, cycle threshold (Ct).
(TIF)
