# Correction: Higher Prevalence and Abundance of *Bdellovibrio bacteriovorus* in the Human Gut of Healthy Subjects

**DOI:** 10.1371/annotation/b08ddcc9-dfdb-4fc1-b2ac-5a4af3051a91

**Published:** 2013-07-17

**Authors:** Valerio Iebba, Floriana Santangelo, Valentina Totino, Mauro Nicoletti, Antonella Gagliardi, Riccardo Valerio De Biase, Salvatore Cucchiara, Lucia Nencioni, Maria Pia Conte, Serena Schippa

In the Introduction section of this article, the authors used several sentences from previous publications without quoting the original sources. The authors would like to apologize for this and acknowledge the original authors of the borrowed text.

The publications involved are the following:

Xu J, Mahowald MA, Ley RE, Lozupone CA, Hamady M, Martens EC, Henrissat B, Coutinho PM, Minx P, Latreille P, Cordum H, Van Brunt A, Kim K, Fulton RS, Fulton LA, Clifton SW, Wilson RK, Knight RD, Gordon JI.

Evolution of symbiotic bacteria in the distal human intestine.

PLoS Biol. 2007 Jul;5(7):e156.

Blaser MJ.

Who are we? Indigenous microbes and the ecology of human diseases.

EMBO Rep. 2006 Oct;7(10):956-60. Review.

Núñez ME, Martin MO, Duong LK, Ly E, Spain EM.

Investigations into the life cycle of the bacterial predator Bdellovibrio bacteriovorus 109J at an interface by atomic force microscopy.

Biophys J. 2003 May;84(5):3379-88.

Intestinal microbiota in inflammatory bowel disease: Friend of foe?

Francesca Fava, Silvio Danese

World J Gastroenterol 2011 February 7; 17(5): 557-566

The sentences which overlap with previous publications, and not quoted within the Introduction, are:

1) The largest collection of microbes resides in our gut, which harbours trillions of bacteria.

Related reference: Xu J, Mahowald MA, Ley RE, Lozupone CA, Hamady M, Martens EC, Henrissat B, Coutinho PM, Minx P, Latreille P, Cordum H, Van Brunt A, Kim K, Fulton RS, Fulton LA, Clifton SW, Wilson RK, Knight RD, Gordon JI. Evolution of symbiotic bacteria in the distal human intestine. PLoS Biol. 2007 Jul;5(7):e156.

2) Although these populations are highly stable, they are still prone to perturbations by environmental insults, with important consequences for our physiology and, consequently, our health.

Related reference: Blaser MJ. Who are we? Indigenous microbes and the ecology of human diseases. EMBO Rep. 2006 Oct;7(10):956-60. Review.

3) In IBD patients, for example, it has been showed a decrease in prevalence of members of the human commensal microbiota (i.e. Clostridium IXa and IV groups Bacteroides, Bifidobacteria) and a concomitant increase in detrimental bacteria (i.e. sulphate-reducing bacteria, Escherichia coli).

Related reference: Intestinal microbiota in inflammatory bowel disease: Friend of foe?

Francesca Fava, Silvio Danese

World J Gastroenterol 2011 February 7; 17(5): 557-566

4) Its life cycle consists of two major steps: a 'free-swimming/gliding' one spent searching for prey in water or soil, and a double 'attack phase/growth' stage spent inside the periplasm of the prey bacterium . Once the resources of the prey have been consumed, the B. bacteriovorus divides into progeny which then lyses the residue cells and swim away to chase new hosts. Depending on the prey concentration and environment, this life cycle takes roughly 3-4 h.

Related reference: Núñez ME, Martin MO, Duong LK, Ly E, Spain EM. Investigations into the life cycle of the bacterial predator Bdellovibrio bacteriovorus 109J at an interface by atomic force microscopy. Biophys J. 2003 May;84(5):3379-88.

The identified issues have no bearing on the results and conclusions of the study. The authors apologize for the instances of plagiarism noted above.

