# Supplementary figures and images for: Higher Prevalence and Abundance of Bdellovibrio bacteriovorus in the Human Gut of Healthy Subjects
Source: PLoS One. 2013 Apr 16;8(4):e61608. doi: 10.1371/journal.pone.0061608 (PMC3628794; doi:10.1371/journal.pone.0061608)

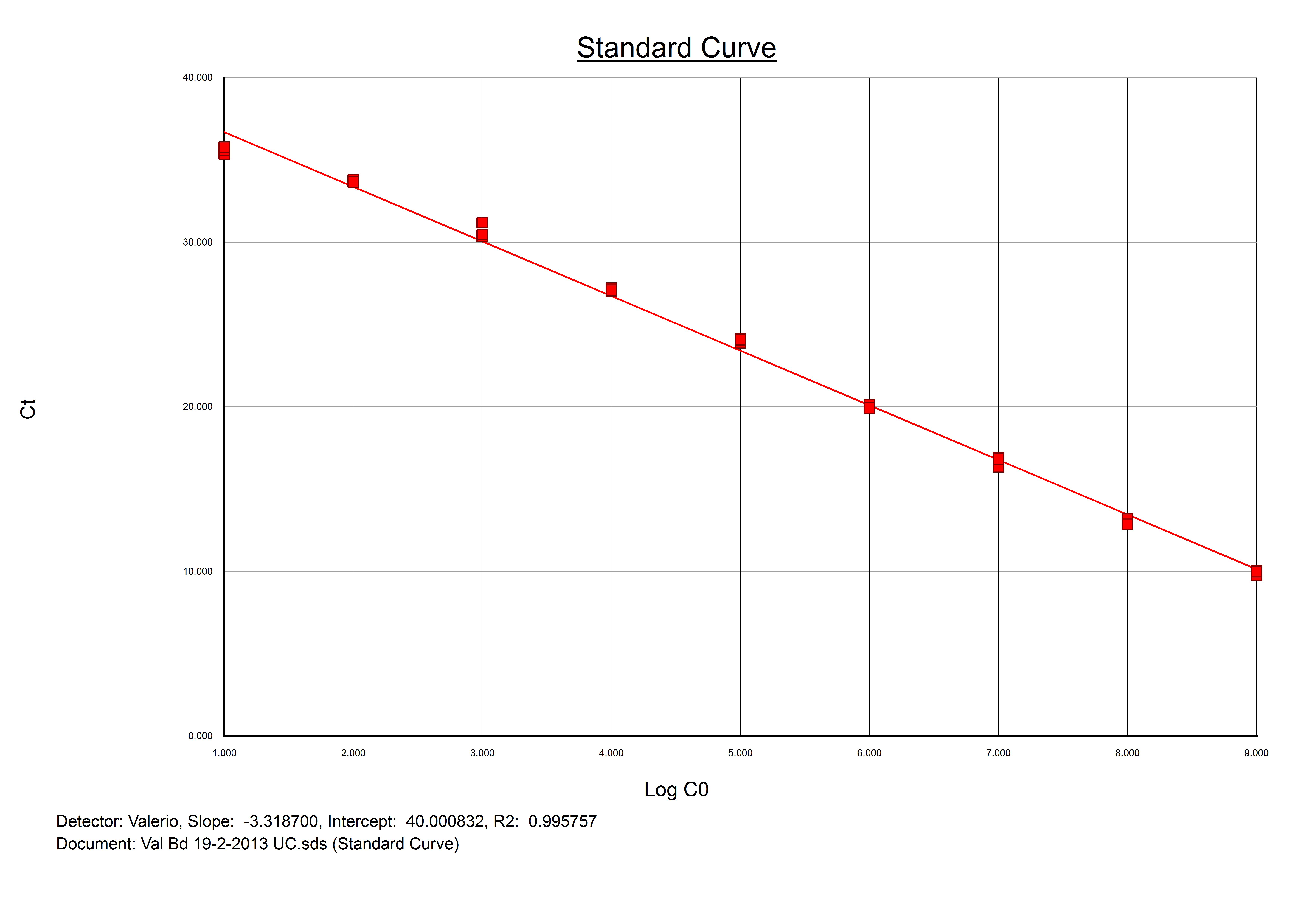

Supplement: Figure S1 — Standard curve for qPCR. Standard curves gave a mean slope of −3.3392±0.0405, a mean intercept of 39.4547±0.3976, and a mean R2 equal to 0.9970±0.0007. As an example, in figure is the standard curve relative to UC samples. On x axis, log of ‘number of plasmid copies’; on y axis, cycle threshold (Ct). (TIF) [file pone.0061608.s001.tif]
